# Clinical prediction rules combining signs, symptoms and epidemiological context to distinguish influenza from influenza-like illnesses in primary care: a cross sectional study

**DOI:** 10.1186/1471-2296-12-4

**Published:** 2011-02-09

**Authors:** Barbara Michiels, Isabelle Thomas, Paul Van Royen, Samuel Coenen

**Affiliations:** 1 Department of Primary and Interdisciplinary Care (Centre for General Practice), University of Antwerp, Universiteitsplein 1, B-2610 Antwerp, Belgium; 2 Scientific Institute of Public Health, National Influenza Centre, Virology, Juliette Wytsmanstraat 14, B-1050 Brussels, Belgium; 3 Laboratory of Medical Microbiology, Vaccine & Infectious Disease Institute (VAXINFECTIO), University of Antwerp, Universiteitsplein 1, B-2610 Antwerp, Belgium

## Abstract

**Background:**

During an influenza epidemic prompt diagnosis of influenza is important. This diagnosis however is still essentially based on the interpretation of symptoms and signs by general practitioners. No single symptom is specific enough to be useful in differentiating influenza from other respiratory infections. Our objective is to formulate prediction rules for the diagnosis of influenza with the best diagnostic performance, combining symptoms, signs and context among patients with influenza-like illness.

**Methods:**

During five consecutive winter periods (2002-2007) 138 sentinel general practitioners sampled (naso- and oropharyngeal swabs) 4597 patients with an influenza-like illness (ILI) and registered their symptoms and signs, general characteristics and contextual information. The samples were analysed by a DirectigenFlu-A&B and RT-PCR tests. 4584 records were useful for further analysis.

Starting from the most relevant variables in a Generalized Estimating Equations (GEE) model, we calculated the area under the Receiver Operating Characteristic curve (ROC AUC), sensitivity, specificity and likelihood ratios for positive (LR+) and negative test results (LR-) of single and combined signs, symptoms and context taking into account pre-test and post-test odds.

**Results:**

In total 52.6% (2409/4584) of the samples were positive for influenza virus: 64% (2066/3212) during and 25% (343/1372) pre/post an influenza epidemic. During and pre/post an influenza epidemic the LR+ of 'previous flu-like contacts', 'coughing', 'expectoration on the first day of illness' and 'body temperature above 37.8°C' is 3.35 (95%CI 2.67-4.03) and 1.34 (95%CI 0.97-1.72), respectively. During and pre/post an influenza epidemic the LR- of 'coughing' and 'a body temperature above 37.8°C' is 0.34 (95%CI 0.27-0.41) and 0.07 (95%CI 0.05-0.08), respectively.

**Conclusions:**

Ruling out influenza using clinical and contextual information is easier than ruling it in. Outside an influenza epidemic the absence of cough and fever (> 37,8°C) makes influenza 14 times less likely in ILI patients. During an epidemic the presence of 'previous flu-like contacts', cough, 'expectoration on the first day of illness' and fever (>37,8°C) increases the likelihood for influenza threefold. The additional diagnostic value of rapid point of care tests especially for confirming influenza still has to be established.

## Background

Especially during an influenza pandemic prompt diagnosis of influenza is important for the individual patient and society as well. Diagnosing of influenza is still essentially based on the interpretation of symptoms and signs, notwithstanding the growing support of point-of-care tests.

All primary care practitioners and especially members of influenza surveillance systems (Fluview(Ilinet), USA: http://www.cdc.gov/flu/, Euroflu, Europe: http://www.euroflu.org) need a performing prediction rule to diagnose influenza. The clinical definitions used for reporting cases of influenza in different surveillance systems[1-3] vary widely, are often imprecise and have never been evaluated[4]. Most frequently inclusion criteria for influenza-like illness (ILI) are based on four to six of the nine criteria (sudden onset, cough, rigors and chills, fever, prostration and weakness, headache, myalgia, widespread aches and pain, influenza in close contact) of the ICHPPC-2 classification (International Classification of Health Problems in Primary Care)[5]. A poor relation between these criteria and laboratory confirmed influenza cases has been reported[6,7].

It is important to distinguish between the classic influenza syndrome consisting of sudden onset, fever, headache, cough, sore throat, myalgia, nasal congestion, weakness and loss of appetite[8], and those symptoms and signs which can be used to discriminate from other ILIs. Besides recurrent symptoms like cough and fever, there are other symptoms like acute onset[8,9], malaise, chills, sore throat, muscle pain and nose symptoms[10,11], that were found in one study, but could not be confirmed in another. Unfortunately these symptoms are frequently seen in other respiratory infections caused by a variety of viral and non-viral pathogens. No single symptom is specific enough to be useful in differentiating influenza from these respiratory infections[10].

Since the development of clinical prediction rules systematically combining symptoms and other information might be a more useful strategy[11], the goal of this study is to formulate a prediction rule for influenza in patients presenting with an ILI with the best diagnostic performance in general practice based on the combination of symptoms, signs and contextual information.

## Methods

### Setting, design and participants

A large cross sectional study was conducted based on the information collected during five consecutive surveillance periods (2002-2007) by the sentinel network of general practitioners (GPs) commissioned by the Scientific Institute of Public Health (SIPH), Brussels in Belgium http://www.iph.fgov.be/epidemio/epien/index10.htm. Age, gender and geographic distribution of the participating GPs are representative for Belgium. Eligible patients were informed about the goal of influenza surveillance, no written informed consent was provided. The information was handled totally anonymously. Ethics approval was granted. Each surveillance period, i.e. from October (week 40) until April (week 20), the sentinel GPs took one oro- and two naso-pharyngeal swabs from some of their patients (all ages) consulting with a new ILI characterized by a broad clinical picture with sudden onset of fever (measurement and threshold undefined), respiratory symptoms (like cough) and systemic symptoms (like myalgia). At the same time they registered the corresponding symptoms and signs as well as general characteristics and contextual information by checking each item if positive on a pre-printed form. Only swab sampled records were included in this study. The swabs were stored in a transport medium (Eagle's minimum essential medium, with addition of antibiotics and antimycotics) before sending them by post (free of charge) to the laboratory of virology of the SIPH National Influenza Centre.

### Test methods

The swabs were tested upon reception using a rapid antigen diagnostic test (DirectigenFlu_A&B). The samples were then submitted to a panel of RT-PCR assays (real time or nested polymerase chain reaction) for typing Influenza A and B, our reference test. All influenza A positive samples were then sub typed (H1N1 and H3N2). Laboratory personal was blinded for the clinical information.

The index tests consisted of a combination of some of the following symptoms or signs collected on the pre-printed form: sudden onset, shivering, weakness, headache, muscle pain, lack of appetite, cough, expectoration, nose-, eye- and ear symptoms, red throat, dyspnoea, rhonchi, gastro-intestinal symptoms, confusion, dizziness, age (years), the number of illness days (from the start of the first ILI symptoms to the day swabs were taken), influenza vaccination, ILI contacts in the family, school- or workplace, highest body temperature (°C) measured before the intake of antipyretics.

### Data management

In the original data file body temperature was dichotomized to below (or equal to) and above 37.8°C. The number of illness days exceeding 14 days were considered as missing.

Extra variables were introduced: 'influenza year', corresponding to the surveillance period each year, starting in October; 'influenza epidemic' corresponding to whether or not the number of ILI consultations exceeds the threshold of 100 cases per 100 000 inhabitants (Source: European Influenza Surveillance Scheme for Belgium[12]); 'RSV (Respiratory Syncytial Virus) epidemic', corresponding to whether or not more than 100 confirmed RSV cases per week were reported by the sentinel laboratories in Belgium[13]. No RSV testing was performed on the swabs.

### Statistical methods

Positively skewed variables were log transformed. The pattern of missing data was considered to be at random (MAR). The variables with missing values (temperature, age and number of illness days) were included in an imputation model with all other symptom variables, influenza epidemic and RSV epidemic. Besides the main effects the interactions were also present in the imputation model, ensuring that interactions could properly be allowed in the analysis models. We conducted 10 imputations using the MCMC (Markov chain Monte Carlo) method (= a single chain for all imputations with 200 burn-in iterations followed by 100 iterations between successive imputations) stratified for the five influenza years, using the multiple imputation procedure of SAS (version 9.2).

Each imputed data set was analysed using a GEE (Generalized Estimating Equations) model with influenza positive PCR as the dependent variable and GP code as a cluster variable (as a check on possible clustering of inclusion criteria and symptom registration within GPs). A backward regression analysis starting from a model with all symptoms and interaction terms (pre-planned on clinical relevance) between all symptoms and influenza epidemic, RSV epidemic, vaccine use, number of illness days and age was performed. When convergence problems occurred the responsible variable was eliminated. After stepwise elimination of interaction terms a forward introduction of interaction terms with borderline p-value was executed. The final model contained all single symptoms, signs and contextual variables together with interaction terms with a p-value less than 0.001 (to deal with multiple comparisons). Parameter estimates of relevant variables and interaction terms were then averaged across data sets by using a bootstrap technique (SAS macro)[14].

Starting from the most relevant variables in the GEE model, we calculated the area under the Receiver Operating Characteristic (ROC) curve (AUC), sensitivity, specificity and likelihood ratios for positive (LR+) and negative test results (LR-) for different single signs, symptoms and context and their combinations taking into account pre-test and post-test odds as described by Janssens A et al[15].

To enforce the internal validity, the outcomes and their 95% confidence interval (CI) were calculated using a bootstrap method. The combination of symptoms, signs and/or context with the best LR+ and LR- were used to define clinical prediction rules taking into account logical clinical order. Finally, sensitivity analyses were done for the different influenza strains A and B, for the different surveillance periods, for different age categories and on the records with complete data.

## Results

**Figure 1:**
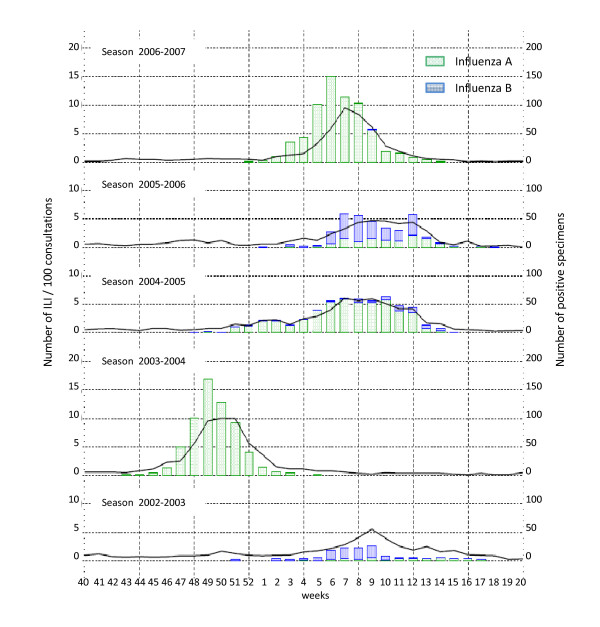
**Number of influenza-like illness (ILI) per 100 consultations and number of influenza positive specimens season 2002/2003 to season 2006/2007**. Note: this figure is part of the historical graphs made by the National Influenza Centre, Virology, Brussels, Belgium http://www.euroflu.org/html/hist_graphs.html and used here with their permission.

In total 138 sentinel general practitioners included and sampled 4 597 of all eligible ILI patients (exact number unknown) during the 5 surveillance periods (Table 1-Figure 1). Information about the general characteristics of the eligible non-participants and reasons for non-participation were not registered. Most records (25.22%) were collected in year 2006-2007. Thirteen records missed data for all signs and symptoms, 18.7% records missed one or more data (maximum three per record; temperature = 14%, age = 2%, illness days = 5%). Through imputing missing data 4584 records could be analysed. No relevant differences were seen between the original, the complete record and the imputed database. In this last database the mean age was 30 (SE 0.28), the mean number of illness days was 1.8 (SE 0.02) and the mean number of positive symptoms was 8.6 (SE 0.04), and 10% were vaccinated against influenza. 70% (3212/4584) of the records were collected during an influenza epidemic and 44% (2036/4584) during an RSV epidemic.

**Table 1:** General characteristics of the included subjects.

	All subjects (n = 4597)	Subjects with full records (n = 3738)	Subjects with full and imputed records (n = 4584)
***Patient age***	(n = 4510)		
Median (range)	28 (0 to 99)	27 (0 to 99)	28 (0 to 99)
Mean (SE)	30.0 (0.29)	29.4 (0.32)	30.0 (0.28)
***Number of subjects (%)***			
By influenza year			
2002-2003	585 (12.73)	479 (12.81)	584 (12.74)
2003-2004	1066 (23.19)	851 (22.77)	1064 (23.21)
2004-2005	996 (21.67)	825 (22.07)	995 (21.71)
2005-2006	785 (17.08)	663 (17.74)	785 (17.12)
2006-2007	1165 (25.34)	920 (24.61)	1156 (25.22)
During an influenza epidemic	3221 (70.07)	2650 (70.89)	3212 (70.07)
With ILI-contact in family. school- or workplace	1492 (32.46)	1195 (31.97)	1490 (32.50)
Vaccinated against influenza	466 (10.14)	377 (10.09)	465 (10.14)
During an RSV epidemic	2039 (44.36)	1635 (43.74)	2036 (44.42)
***Number of symptoms***			
Median (range)	8 (0 to 18)	9 (1 to 18)	9 (1 to 18)
Mean (SE)	8.4 (0.04)	8.6 (0.04)	8.6 (0.04)
***Number of illness days^a^***	(n = 4381)		
Median (range)	1.5 (0 to 39)	1.5 (0 to 14)	2 (0 to 14)
Mean (SE)	1.76 (0.02)	1.72 (0.02)	1.77 (0.02)
*Number of records per GP*	(n = 138)	(n = 134)	(n = 137)
Median (range)	10 (1 to 247)	7 (1 to 217)	10 (1 to 246)
Mean (SE)	33.5 (4.5)	27.9 (4.0)	33.5 (4.5)

SE = standard error

^a ^illness days above 14 days were considered as missing

52.6% of the swabs were found positive for influenza on RT-PCR, which corresponds to a pre-test odds of 1.11 (adjusted by bootstrapping to 1.01 (95%CI 0.94-1.08)). The final GEE model contained all the variables recorded and defined except confusion. It was eliminated because of convergence problems. Only two interaction terms were withheld: influenza epidemic*ILI contacts and expectoration*illness days.

**Figure 2:**
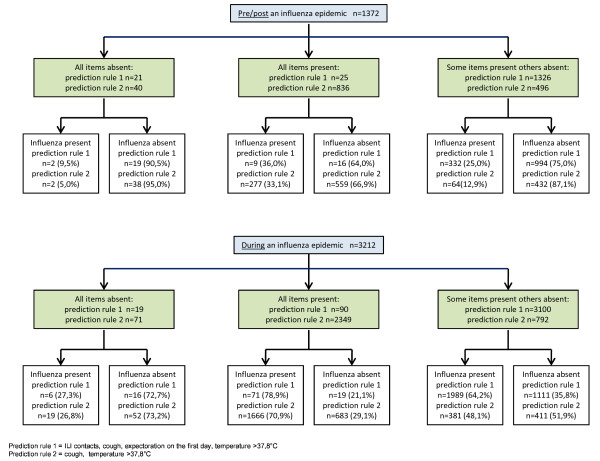
**Diagnostic flow-diagram pre/post and during the influenza epidemics n = 4584 (imputed data)**. Note: about 30% of all the ILI patients were swabbed. No information is available about the no swabbed ILI patients. From the 4597 swabbed patients 13 records missed any clinical information. Of the remaining 4584, 18.7% had missing values in at least one of the following items: age, number of illness days and/or temperature, 3738 were full records.

During an influenza epidemic 64% (2066/3212) of the records were positive for influenza compared with 25% (343/1372) before or after (Figure 2). Besides influenza epidemic other important predictors of influenza cases were no vaccination, body temperature above 37.8°C, cough, nose symptoms and expectoration on the first day of illness (Table 2). ILI contacts were more predictive pre/post an epidemic (ORadj 3.14 (2.23-4.05)) than during an epidemic (ORadj 1.24 (1.03-1.44)). Age and many symptoms such as sudden onset, shivering, weakness, headache, muscle pain, lack of appetite and eye symptoms were also no longer significant in the adjusted full model. There was no difference between the two groups for ear symptoms, red throat, dyspnoea, rhonchi, gastro-intestinal problems, confusion and dizziness.

**Table 2:** Presence of context, signs and symptoms in influenza positive and negative subjects and corresponding crude^a ^and adjusted odds ratios (95%CI)^b^.

Contexts. signs and symptoms	Influenza pos (n = 2409)	Influenza neg (n = 2175)	Crude OR (95%CI)	Adjusted OR (95%CI)
Body temperature >37.8°C. No. (%)	2271 (94.3)	1902 (87.5)	2.36 (1.91 to 2.92)	2.13 (1.58 to 2.68)^c^
Sudden onset. No. (%)	2154 (89.4)	1874 (86.2)	1.36 (1.14 to 1.62)	1.25 (0.99 to 1.51)
Influenza epidemic. No. (%)	2066 (85.8)	1146 (52.7)	5.41 (4.69 to 6.23)	6.28 (5.03 to 7.54)^c^
Cough. No. (%)	2060 (85.5)	1425 (65.5)	3.11 (2.69 to 3.59)	2.59 (2.14 to 3.04)^c^
Shivering. No. (%)	1999 (83.0)	1706 (78.4)	1.34 (1.16 to 1.55)	1.13 (0.92 to 1.33)
Weakness. No. (%)	1965 (81.6)	1691 (77.7)	1.27 (1.10 to 1.46)	1.03 (0.84 to 1.21)
Headache. No. (%)	1918 (79.6)	1590 (73.1)	1.44 (1.25 to 1.65)	1.16 (0.96 to 1.37)
Muscle pain. No. (%)	1838 (76.3)	1567 (72.0)	1.25 (1.09 to 1.43)	1.16 (0.97 to 1.36)
Nose symptoms. No. (%)	1620 (67.2)	1208 (55.5)	1.64 (1.46 to 1.85)	1.38 (1.18 to 1.58)
Red throat. No. (%)	1529 (63.5)	1333 (61.3)	1.10 (0.97 to 1.24)	0.95 (0.81 to 1.09)
Lack of appetite. No. (%)	1453 (60.3)	1130 (52.0)	1.41 (1.25 to 1.58)	1.15 (0.98 to 1.32)
RSV epidemic. No. (%)	1026 (42.6)	1010 (46.4)	0.86 (0.76 to 0.96)	1.14 (0.99 to 1.29)
ILI contacts in the family. school or workplace. No. (%)	928 (38.5)	562 (25.8)	1.80 (1.59 to 2.04)	Not in model
ILI contacts pre/post an epidemic. No. (%). n = 1372	134 (39.1)	180 (17.5)	3.02 (2.31 to 3.96)	3.14 (2.23 to 4.05)^c^
ILI contacts during an epidemic. No. (%)^c^. n = 3212	79'4 (38.4)	382 (33.3)	1.25 (1.07 to 1.45)	1.24 (1.03 to 1.44)
Expectoration. No. (%)	661 (27.4)	456 (21.0)	1.43 (1.24 to 1.63)	Not in model
Expectoration on illness day 0. No. (%). n = 117	9 (18.37)	15 (22.06)	0.80 (0.32 to 2.00)	2.24 (0.93 to 3.55)
Expectoration on illness day 1. No. (%). n = 2101	269 (25.33)	165 (15.88)	1.80 (1.45 to 2.23)	1.36 (1.06 to 1.66)^c^
Expectoration on illness day 2. No. (%). n = 1676	262 (27.61)	159 (21.87)	1.35 (1.09 to 1.71)	1.00 (0.83 to 1.17)
Eye symptoms. No. (%)	659 (27.4)	481 (22.1)	1.33 (1.16 to 1.52)	1.08 (0.90 to 1.27)
Gastro-intestinal symptoms. No. (%)	368 (15.3)	374 (17.2)	0.87 (0.74 to 1.02)	0.84 (0.68 to 1.00)
Ear symptoms. No. (%)	355 (14.7)	304 (14.0)	1.06 (0.90 to 1.26)	0.88 (0.71 to 1.05)
Dyspnoe. No. (%)	236 (9.8)	186 (8.6)	1.16 (0.95 to 1.42)	1.18 (0.90 to 1.45)
Dizziness. No. (%)	233 (9.7)	177 (8.1)	1.21 (0.99 to 1.48)	1.18 (0.87 to 1.48)
Vaccination against influenza. No. (%)	198 (8.2)	267 (12.3)	0.64 (0.53 to 0.78)	0.67 (0.50 to 0.85)
Ronchi. No. (%)	166 (6.9)	136 (6.3)	1.11 (0.88 to 1.40)	0.93 (0.65 to 1.20)
Confusion. No. (%)	22 (0.9)	22 (1.0)	0.90 (0.50 to 1.63)	not in model
Age difference of 10 years	NA	NA	NA	0.97 (0.94 to 1.01)
Age. mean (SE) ^c^	28.90 (0.39)	31.27 (0.41)	NA	Not in model
Temp. mean (SE) ^c ^	38.70 (0.01)	38.46 (0.02)	NA	Not in model

NA = not applicable CI = confidence interval

^a ^Crude Odds ratios were estimated using Chi^2^

^b ^Adjusted odds ratios were estimated using Generalized Estimating Equations. 95%CI were calculated by bootstrap after multiple imputation.

^c ^significant different: p-value < 0.0001

The variables influenza epidemic, ILI contacts, cough, expectoration, body temperature >37.8°C and the interaction terms 'ILI contacts'*epidemic and expectoration*'number of illness days' all had a p-value < 0,0001 in the multivariate model. Influenza epidemic, ILI contacts, cough, 'expectoration per illness day', nose symptoms, lack of appetite and 'body temperature > 37.8°C' are the most performing symptoms to discriminate influenza from other ILIs according the AUROC (Table 3). Starting with influenza epidemic and stepwise adding ILI contacts, cough, body temperature and expectoration per day (prediction rule 1) or adding cough and body temperature (prediction rule 2) both give a final AUC of 0.75 (0.73-0.76). Adding more variables does not raise the AUC any further.

**Table 3:** Area under the Receiver Operating Characteristic curve (95% CI) of symptoms, signs and context, single or in combination, for the diagnosis of influenza.

Symptoms. signs and context	Single	Combinations					
Influenza epidemic	**0.67 **(0.65 to 0.68)	Influenza epidemic +	Influenza epidemic +	Influenza epidemic +	Influenza epidemic +	Influenza epidemic +	Influenza epidemic +
ILI contacts	0.56 (0.55 to 0.58)	0.68 (0.67 to 0.70)	ILI contacts^a ^+	ILI contacts +	ILI contacts +	0.72 (0.71-0.74)	0.74 (0.72-0.75)
Cough	0.60 (0.59 to 0.61)	**0.71 **(0.70 to 0.72)	**0.72 **(0.71 to 0.74)	Cough +	Cough +	Cough+	Cough+
Temp > 37.8°C	0.54 (0.53 to 0.55)	0.69 (0.68 to 0.70)	0.71 (0.69 to 0.72)	**0.74 **(0.72 to 0.75)	Temp > 37.8°C +	**0.75 **(0.73-0.76)	Temp+
Expectoration*illness day^b^	0.56 (0.55 to 0.58)	0.70 (0.68 to 0.71)	0.70 (0.69 to 0.72)	0.73 (0.72 to 0.75)	**0.75 **(0.73 to 0.76)	0.72 (0.71 to 0.74)	**0.75 **(0.73 to 0.76)
Lack of appetite	0.54 (0.53 to 0.56)	0.69 (0.67 to 0.70)	0.70 (0.68 to 0.71)	0.73 (0.72 to 0.74)	0.74 (0.73 to 0.76)	0.72 (0.71 to 0.74)	0.75 (0.73 to 0.76)
Nose symptoms	0.56 (0.54 to 0.57)	0.69 (0.68 to 0.71)	0.70 (0.69 to 0.72)	0.73 (0.71 to 0.74)	0.74 (0.73 to 0.76)	0.70 (0.69 to 0.72)	0.75 (0.74 to 0.76)
Expectoration	0.53 (0.52 to 0.54)	0.68 (0.67 to 0.69)	0.69 (0.68 to 0.71)	0.72 (0.71 to 0.74)	0.74 (0.72 to 0.75)	0.71 (0.70 to 0.73)	0.75 (0.73 to 0.76)
Muscle pain	0.52 (0.51 to 0.53)	0.68 (0.66 to 0.69)	0.69 (0.68 to 0.71)	0.73 (0.71 to 0.74)	0.74 (0.73 to 0.76)	0.72 (0.70 to 0.73)	0.75 (0.73 to 0.76)
Flu 'vaccine	0.52 (0.51 to 0.53)	0.67 (0.66 to 0.69)	0.69 (0.67 to 0.70)	0.73 (0.71 to 0.74)	0.74 (0.72 to 0.75)	0.72 (0.70 to 0.73)	0.75 (0.73 to 0.76)
Vertigo	0.51 (0.50 to 0.52)	0.67 (0.66 to 0.68)	0.69 (0.67 to 0.70)	0.73 (0.71 to 0.74)	0.74 (0.73 to 0.75)	0.71 (0.70 to 0.73)	0.75 (0.73 to 0.76)

During an influenza epidemic variables with high sensitivity such as cough (0.82 (0.81-0.84)) and body temperature (0.91 (0.89-0.92)) have a poor specificity (respectively 0.39 (0.36-0.42) and 0.19 (0.16-0.22)) (Table 4). During the pre/post epidemic period specific variables such as ILI contact (0.82 (0.80-0.85)), 'expectoration on the first day' (0.92 (0.65-1.00) have a bad corresponding sensitivity (0.42 (0.36-0.47) and 0.11 (0.00-0.42), respectively).

**Table 4:** The sensitivity, specificity, likelihood ratios of the positive (LR+) and negative test result (LR-) of single and combined symptoms, signs and context for the diagnosis of influenza (95%CI)^a^.

Symptoms. signs and context	Sensitivity (95% CI)	Specificity (95% CI)	LR+ (95% CI)	Cumulative ^b^ LR+ (95% CI)	LR- (95% CI)	Cumulative ^b^ LR- (95% CI)
Influenza epidemic	0.88 (0.86 to 0.89)	0.42 (0.39 to 0.44)	1.50 (1.43 to 1.57)		0.30 (0.26 to 0.33)	
*During an epidemic*			*1.50 (1.43 to 1.57)*		*1.50 (1.43 to 1.57)*	
Prediction rule 1						
+ILI contact	0.42 (0.38 to 0.46)	0.63 (0.58 to 0.68)	1.12 (1.01 to 1.24)	1.68 (1.49 to 1.88)	0.93 (0.87 to 0.98)	1.38 (1.28 to 1.49)
+cough	0.81 (0.79 to 0.84)	0.40 (0.36 to 0.44)	1.35 (1.29 to 1.40)	2.27 (2.01 to 2.53)	0.47 (0.42 to 0.52)	0.65 (0.56 to 0.74)
+expectoration day1	0.10 (0.00 to 0.40)	0.93 (0.67 to 1.00)	1.30 (1.08 to 1.53)	2.96 (2.38 to 3.54)	0.93 (0.86 to 1.00)	0.54 (0.40 to 0.68)
+body temp > 37.8°C	0.90 (0.87 to 0.92)	0.21 (0.16 to 0.26)	1.13 (1.08 to 1.19)	3.35 (2.67 to 4.03)	0.49 (0.41 to 0.58)	0.26 (0.18 to 0.34)
Prediction rule 2						
+ cough	0.82 (0.81 to 0.84)	0.39 (0.36 to 0.42)	1.34 (1.29 to 1.39)	2.01 (1.90 to 2.13)	0.46 (0.41 to 0.51)	0.69 (0.60 to 0.78)
+body temp > 37.8°C	0.91 (0.89 to 0.92)	0.19 (0.16 to 0.22)	1.12 (1.08 to 1.16)	2.26 (2.11 to 2.40)	0.49 (0.40 to 0.58)	0.34 (0.27 to 0.41)
*Pre/post an epidemic*			*0.30 (0.26 to 0.33)*		*0.30 (0.26 to 0.33)*	
Prediction rule 1						
+ ILI contact	0.42 (0.36 to 0.47)	0.82 (0.80 to 0.85)	2.36 (1.93 to 2.79)	0.69 (0.54 to 0.85)	0.71 (0.64 to 0.78)	0.21 (0.18 to 0.24)
+cough	0.81 (0.78 to 0.85)	0.40 (0.34 to 0.46)	1.35 (1.28 to 1.42)	0.93 (0.73 to 1.14)	0.47 (0.41 to 0.53)	0.11 (0.09 to 0.13)
+expectoration day1	0.11 (0.00 to 0.42)	0.92 (0.65 to 1.00)	1.30 (1.08 to 1.53)	1.21 (0.89 to 1.54)	0.93 (0.85 to 1.00)	0.09 (0.06 to 0.11)
+body temp > 37.8°C	0.92 (0.88 to 0.95)	0.17 (0.11 to 0.23)	1.11 (1.05 to 1.16)	1.34 (0.97 to 1.72)	0.48 (0.39 to 0.57)	0.04 (0.03 to 0.05)
Prediction rule 2						
+cough	0.78 (0.76 to 0.81)	0.45 (0.42 to 0.48)	1.42 (1.37 to 1.47)	0.42 (0.38 to 0.47)	0.49 (0.43 to 0.54)	0.14 (0.12 to 0.17)
+body temp > 37.8°C	0.93 (0.91 to 0.95)	0.15 (0.12 to 0.17)	1.09 (1.06 to 1.12)	0.46 (0.41 to 0.51)	0.48 (0.39 to 0.57)	0.07 (0.05 to 0.08)

CI = confidence interval

^a ^analysis was performed on the database with full and imputed records n = 4584

^b ^cumulative = combined diagnostic value of all the symptoms. signs or context taken into account so far

In general the LR- of the different symptoms performs better than the LR+. The values are alike during and pre/post an influenza epidemic, except for ILI contacts because of the significant interaction of this variable with influenza epidemic.

The LR+ of an influenza epidemic is 1.50 (1.43-1.57). Prediction rule 1 then gives a cumulative LR+ of 3.35 (2.67-4.03) and an LR- of 0.26 (0.18-0.34). Prediction rule 2 gives a cumulative LR+ of 2.26 (2.11-2.40) and an LR- of 0.34 (0.27-0.41). Adding more information has no additional diagnostic value.

The LR- of an influenza epidemic is 0.30 (0.26-0.33). In this situation 'previous ILI contacts' is more important and this information raises the likelihood by a factor of 2.36. For prediction rule 1 the cumulative LR+ now is 1.34 (0.97-1.72). The corresponding LR- is 0.04 (0.03-0.05). For prediction rule 2 the LR+ now is 0.46 (0.41-0.51), the LR- is 0.07 (0.05-0.08). When some symptoms are present and others are absent the prediction rules have lower LR+ and higher LR-.

There is no statistical or relevant difference in performance of both prediction rules between influenza A and B, nor between the surveillance periods or ages (Table 5). Pre/post an influenza epidemic no prediction rule can help to confirm influenza in the age group <5 years and >65 years. Ruling out influenza seems to be easier in the younger age groups.

**Table 5:** Likelihood ratios of the positive (LR+) and negative test result (LR-) of two prediction rules^a ^in subgroups of the imputed original database and in the full record database (95%CI).

			During influenza epidemic		Pre/post an influenza epidemic	
	Prediction rule^a^	n	LR+	LR-	LR+	LR-
Full record analysis	1	3738	3.65 (1.76 to 5.54)	0.23 (0.08 to 0.37)	1.23 (0.34 to 2.11)	0.04 (0.01 to 0.06)
	2		2.24 (1.95 to 2.54)	0.27 (0.12 to 0.41)	0.43 (0.33 to 0.54)	0.05 (0.02 to 0.08)
Influenza A	1	4176	3.43 (2.69 to 4.18)	0.30 (0.20 to 0.40)	1.46 (1.05 to 1.87)	0.05 (0.03 to 0.06
	2		2.21 (2.06 to 2.36)	0.33 (0.26 to 0.41)	0.46 (0.40 to 0.51)	0.07 (0.05 to 0.09)
Influenza B	1	2566	5.05 (3.75 to 6.36)	0.13 (0.07 to 0.19)	1.16 (0.64 to 1.68)	0.01 (0.00 to 0.02)
	2		4.01 (3.68 to 4.33)	0.34 (0.20 to 0.48)	0.49 (0.37 to 0.61)	0.04 (0.02 to 0.07)
Influenza year (2002 - 2003)	1	584	3.73 (0.63 to 6.83)	0.13 (0.00 to 0.32)	1.79 (0.19 to 3.39)	0.06 (0.00 to 0.13)
	2		2.28 (1.72 to 2.83)	0.22 (0.00 to 0.45)	1.08 (0.84 to 1.32)	0.10 (0.01 to 0.19)
Influenza year (2003 - 2004)	1	1064	4.00 (0.49 to 7.51)	0.13 (0.04 to 0.21)	1.72 (0.00 to 4.10)	0.02 (0.01 to 0.03)
	2		2.19 (1.87 to 2.51)	0.25 (0.15 to 0.36)	0.36 (0.24 to 0.47)	0.04 (0.02 to 0.06)
Influenza year (2004 - 2005)	1	995	3.93 (2.07 to 5.78)	0.32 (0.13 to 0.51)	3.03 (1.64 to 4.42)	0.07 (0.03 to 0.10)
	2		2.52 (2.16 to 2.88)	0.42 (0.23 to 0.62)	0.79 (0.65 to 0.93)	0.13 (0.07 to 0.19)
Influenza year (2005 - 2006)	1	785	2.90 (1.03 to 4.77)	0.40 (0.05 to 0.74)	0.65 (0.28 to 1.02)	0.06 (0.02 to 0.11)
	2		2.90 (2.47 to 3.33)	0.61 (0.29 to 0.92)	0.50 (0.40 to 0.60)	0.10 (0.06 to 0.15)
Influenza year (2006 - 2007)	1	1156	2.04 (1.19 to 2.90)	0.33 (0.09 to 0.57)	1.07 (0.39 to 1.75)	0.04 (0.01 to 0.08)
	2		1.79 (1.59 to 2.00)	0.24 (0.13 to 0.35)	0.38 (0.27 to 0.50)	0.05 (0.02 to 0.08)
Age < 5 years	1	267	2.70 (0.00 to 6.02)	0.06 (0.00 to 0.82)	0.69 (0.00 to 2.24)	0.01 (0.00 to 0.11)
	2		1.58 (1.10 to 2.05)	0.12 (0.00 to 1.12)	0.25 (0.08 to 0.42)	0.02 (0.00 to 0.17)
Age 5 to 14 years	1	931	3.57 (1.08 to 6.07)	0.14 (0.00 to 0.27)	1.43 (0.25 to 2.61)	0.03 (0.00 to 0.05)
	2		1.91 (1.65 to 2.16)	0.22 (0.02 to 0.42)	0.44 (0.32 to 0.56)	0.05 (0.00 to 0.10)
Age 15 to 64 years	1	3137	3.77 (2.69 to 4.85)	0.31 (0.22 to 0.41)	1.71 (1.09 to 2.34)	0.05 (0.03 to 0.07)
	2		2.29 (2.10 to 2.48)	0.32 (0.24 to 0.41)	0.48 (0.42 to 0.54)	0.07 (0.05 to 0.09)
Age > 64 years	1	249	5.31 (0.00 to 20.43)	0.39 (0.00 to 1.01)	0.67 (0.00 to 2.91)	0.14 (0.00 to 0.29)
	2		1.68 (0.91 to 2.44)	0.24 (0.00 to 0.73)	0.46 (0.26 to 0.66)	0.07 (0.00 to 0.16)

CI = confidence interval

^a ^Prediction rule 1 = influenza epidemic + previous ILI contact + cough + expectoration on day1 + body temp > 37.8°C; Prediction rule 2 = influenza epidemic + cough + body temp > 37.8°C

## Discussion

In patients presenting with ILI in primary care ruling out influenza is easier than confirming it. Pre/post an influenza epidemic the absence of cough and fever (>37.8°C) lowers a pre-test probability of 25% to a post-test probability of 7%. During an epidemic with a pre-test probability of 62%, the absence of these symptoms gives a post-test probability of 27%. To confirm influenza the presence of previous ILI contacts', cough, 'expectoration on the first illness day' combined with 'fever >37.8°C' results in a post-test probability of 79%. Pre/post an epidemic the presence of these items gives a post-test probability of 60%.

Our study had to deal with some limitations. Our study was not designed to evaluate the additional value of rapid point of care tests, which were only performed in the virology laboratory and not at the GP practice.

Only 20,7% of the records mentioned expectoration on the first day of illness. Normally influenza is defined as a respiratory infection with a dry cough. So this symptom was only helpful in a minority of cases in the confirmation of influenza, but even when absent prediction rule 1 is still quite useful with a LR+ of 2.38 during an influenza epidemic.

Gender information is missing in our study, but until now no difference has been described between males and females in the symptomatology of influenza.

Sentinel GPs did not include every patient with ILI. The choices they made and the reasons for them are unclear: sometimes the number of swabs were restricted by the virology lab and patients could refuse to participate. There is no reason to believe that a systematic selection bias took place. Especially patients with higher fever are included by the sentinel GPs and this must be kept in mind, when extrapolating our results. The youngest age group (<5 years) is under-represented in our database. Probably because it is not easy to take swabs from small children and/or in Belgium parents could have chosen to go directly to a paediatrician for their first consult. Especially in this age group extra validation is required. There are a smaller amount of samples in the older (>64 years) age group, but this is merely due to the lower incidence of influenza in this age group.

The advantage of our study is the large number of records over five surveillance periods. This allows an extensive analysis and robust results. An advanced statistical approach was adopted to deal with missing data other than the outcome variable to correct for potential biases or overestimation of diagnostic values and multiple comparisons. We also took into account the influence of symptoms, signs or context already considered on the diagnostic values of new items added as well as interactions.

That expectoration is only important when occurring during the first few days has never been considered in other studies [Additional file 1]. Carrat et al[4] found expectoration to be present more frequently among influenza A positive patients. Loda[16], describing the symptoms of volunteers with an influenza illness after nasal inoculation by a wild type influenza A, found that cough, rhonchi and expiratory fine rales were the most frequent and persistent manifestations. Of the initial 426 cases of the 2009 pandemic influenza A (H1N1) cases 104 (24.5%) suffered from sputum production on admission in hospital[17]. This percentage is comparable with the incidence of expectoration on the first day of illness in influenza cases in our study (25.3%).

The number of illness days up to now has never been tested in interaction with other variables and was seldom considered as a continuous variable. Stein et al[18] compared the performance of clinician judgement, a rapid influenza test and the prediction rule cough and fever, and did not see a significant effect of the duration of illness on the overall accuracy of the latter prediction rule. This is confirmed by our findings. In addition, the diagnostic value of symptoms and signs outside epidemics is scarce in the literature. To date, the value of information about previous contact with other ILI cases, especially pre/post the epidemic, has never been shown. The prevailing prediction rule has been generated from a selected patient population that was recruited to study the effects of neuraminidase inhibitors. The strict inclusion criteria for those studies excluded many patients that would have normally presented for evaluation of acute respiratory symptoms in primary care[18].

During an influenza epidemic our findings about cough and fever especially, corroborate previous findings. Boivin et al concluded in 2000 that the combination of cough, fever and the knowledge of an epidemic gave the best prediction and that physicians could correctly diagnose influenza in over 60-70% of their patients on the basis of clinical symptoms alone[19]. The systematic review of Call et al[10], including the large study of Monto[8], showed that no symptom or sign had an LR+ greater than 2 in studies that enrolled patients with disregard to age. To rule out influenza the absence of fever (LR- 0.40; 95%CI: 0.25-0.66), cough (LR- 0.42; 0.31-0.57) or nasal congestion (LR- 0.49; 0.42-0.59) were the only findings that had an LR- less than 0.5.

In our study we found small, not statistically significant, differences in diagnostic accuracy of the two prediction rules according to different age-categories, and no statistically significant interaction between the individual variables and age in the multivariate model. In the study of Carrat[4], with a smaller sample size, this was also the case. Govaerts et al[9] concluded that fever and cough (and acute onset) give the best prediction in a population of 60+ elderly during an influenza season (without pre-selection). The different symptom patterns for different strains, found by Carrat[4], could not be confirmed by Monto[8,10]. We found a significantly different LR+ for cough and fever between influenza A and B, but the clinical significance of this finding is limited.

The derivation and part of the validation[20,21] have been achieved for prediction rule 1. Prediction rule 2 has previously been mentioned in the literature[18,19] and is now broadly validated in our study. A large prospective diagnostic study for influenza taking into account our remarks might generate the broad validation and impact analysis necessary to successfully implement our findings.

## Conclusions

In patients presenting with an influenza-like illness to primary care, the asymmetric diagnostic values of combinations of clinical and contextual information, i.e. ruling out is easier than ruling in, have important implications for the management of influenza. Outside an epidemic, influenza is easily ruled out by the absence of cough and fever. Clinical and contextual information alone might not be sufficient to rule in influenza and to make treatment decisions, although 'expectoration on the first day of illness' combined with 'previous flu-like contacts', cough and fever (>37,8°C) increases the likelihood of influenza threefold during an epidemic. The place and the additional diagnostic value of rapid point of care tests on top of clinical and contextual information still has to be established.

## Competing interests

The authors declare that they have no competing interests.

## Authors' contributions

BM initiated and designed the study, obtained funding, was involved in analyzing and interpreting the data and in writing the manuscript. IT was responsible for data collection and was involved in revising the manuscript. SC and PVR were involved in analyzing and interpreting data and in revising the manuscript. All authors had full access to all of the data (including statistical reports and tables) in the study and can take responsibility for the integrity of the data and the accuracy of the data analysis and have seen and approved the final manuscript.

## Pre-publication history

The pre-publication history for this paper can be accessed here:

http://www.biomedcentral.com/1471-2296/12/4/prepub

## Supplementary Material

Additional file 1 **literature compilation regarding influenza diagnosis**. Additional file with information about previous published prediction rules and diagnostic accuracy studies. Click here for file
