# Supplementary material for: Clinical prediction rules combining signs, symptoms and epidemiological context to distinguish influenza from influenza-like illnesses in primary care: a cross sectional study
Source: BMC Fam Pract. 2011 Feb 9;12:4. doi: 10.1186/1471-2296-12-4 (PMC3045895; doi:10.1186/1471-2296-12-4)
Supplement: Additional file 1 — literature compilation regarding influenza diagnosis. Additional file with information about previous published prediction rules and diagnostic accuracy studies. [file 1471-2296-12-4-S1.DOCX]

***Previous published prediction rules and diagnostic accuracy studies***

*Comparison is difficult because of differences in reference diagnostic test or inclusion criteria: other studies are mostly performed only during an epidemic, in an hospital setting and/or within 72 hours after onset of illness. In short following results were previously published:*

*Call JAMA 2005(1) systematic review:*

*No symptom or sign had a summary LR greater than 2 in studies that enrolled patients without regard to age. For decreasing the likelihood of influenza, the absence of fever (LR, 0.40; 95%CI, 0.25-0.66), cough (LR, 0.42; 95% CI, 0.31-0.57), or nasal congestion (LR, 0.49; 95% CI, 0.42-0.59) were the only findings that had summary LR less than 0.5. In studies limited to patients aged 60 years or older, the combination of fever, cough, and acute onset (LR, 5.4; 95% CI, 3.8-7.7), fever and cough (LR, 5.0; 95% CI, 3.5-6.9), fever alone (LR, 3.8; 95% CI, 2.8-5.0), malaise (LR, 2.6; 95% CI, 2.2-3.1), and chills (LR, 2.6; 95% CI, 2.0-3.2) increased the likelihood of influenza to the greatest degree. The presence of sneezing among older patients made influenza less likely (LR, 0.47; 95% CI, 0.24-0.92).*

*Ebell AFP 2004(2) systematic review;*

*Epidemiology of* ***seasonal influenza epidemic****: Rigors LR+=7.2, combination of fever and presenting within 3 days of the onset of illness LR+=3 were best at ruling in influenza when present. Ruling out influenza: any systemic symptom LR-=0,36, coughing LR-=0.38, not being able to cope with daily activities (LR-0.39) and being confined to bed (LR-0.50). Cough, nasal congestion and fever (subjective or objective)had the highest calculable areas under the ROC curve: range 0,679 (cough) to 0,653(objective temp)*

*Stein AEM 2005(3) n=53 influenza positive/n=205 influenza negative; emergency department,* ***no distinction between epidemic and pre and/or post-epidemic period***

*Clinician judgement had no clinically significant difference in the accuracy of influenza diagnosis compared to the prediction rule; cough and fever: sens 40% (27 – 54%), spec 92% (87 – 95%), LRpos 5,1 (2,7 – 9,6), LRneg 0,7 (0,5 – 0,8)*

*The investigators didn’t see a significant effect of duration of illness on the overall accuracy of the clinical decision rule (fever and cough) or the rapid influenza test.*

*In general a low sensitivity and a high specificity was found for all diagnostic methods (= clinician judgement, rapid influenza test, decision rule=cough and fever) in a general population of adults presenting with acute respiratory illness. They were unable to demonstrate any significant improvement in clinical decision making using a decision rule or rapid test*

*Walsh et al. JAGS 2002(4): n=61 Influenza + 271 Non-Influenza cases*

*Value of clinical parameters in predicting influenza in older persons and those with underlying cardiopulmonary conditions hospitalized with respiratory illnesses n=332 (61 influenza confirmed): neither single clinical parameters nor logistic regression analysis using all parameters clearly discriminated influenza from non-influenza cases. The complex of cough, temperature of 38°C or higher and illness duration of 7 days or less provided the best discrimination between infected and uninfected subjects* ***during the epidemic*** *period (sensitivity = 78%;specificity=73%)*

*Zambon AIM 2001(5)n=791 Influenza + 242 Non-Influenza cases; community – during an* ***epidemic***

*Baseline temperature and the severity of cough and fever showed a significant positive correlation with the number of positive test results (all p< 0,001)and sore throat showed a significant negative correlation (p=0,01). Vaccination status should not automatically exclude the diagnosis of influenza (of the vaccinated patients included in the neuraminidase trials 80% had influenza as severe as that recorded in nonvaccinated patients)*

*Boivin CID 2000(6) n= 79 Influenza + 21 Non-Influenza; hospital* ***during an epidemic***

*The combination of cough and fever was associated with an OR of 6.7 (1.4 – 34.1) (sensitivity=77.6% - specificity = 55.0%)*

*Monto AIM 2000(7) n=2470 influenza pos+ 1274 non-influenza cases; during an* ***epidemic***

*Cough and fever are good predictors of infection among patients with an influenza like illness when influenza is present in the community: PPV=79%; p<0,001.*

*Carrat CID 1999(8) n= 158 Influenza + 442 Non-Influenza* ***during an influenza epidemic****;*

*The frequency of chills, moderate to severe fatigue, headache, sneezing, cough, thoracic pain when taking a deep breath, rhinorhea, expectoration of sputum and lacrimation or conjunctival injection was significantly greater among influenza A positive patients.*

*Multivariate analysis identified a temperature of >38,2°C and rhinorhea as clinical predictors.*

*Lack of difference or trend in the predictive values with age, the rate is different, H1N1 more in younger people, but clinical picture did not differ significantly with age.*

*Govaert Fam pract 1998(9)n=121 Influenza + 1717 Non-Influenza cases; family practices*

*Fever and cough (and acute onset) gives the best prediction in a population of 60+ elderly* ***during an influenza season*** *(without preselection)*

1. *Call SA, Vollenweider MA, Hornung CA, Simel DL, McKinney WP. Does this patient have influenza? JAMA. 2005;293(8):987-997*
2. *Ebell MH, White LL, Casault T. A systematic review of the history and physical examination to diagnose influenza. J Am Board Fam Pract. 2004;17(1):1-5.*
3. *Stein J, Louie J, Flanders S, et al. Performance characteristics of clinical diagnosis, a clinical decision rule, and a rapid influenza test in the detection of influenza infection in a community sample of adults. Ann Emerg Med. 2005;46(5):412-419.*
4. *Walsh EE, Cox C, Falsey AR. Clinical features of influenza A virus infection in older hospitalized persons. J Am Geriatr Soc. 2002;50(9):1498-1503.*
5. *Zambon M, Hays J, and Webster A, Diagnosis of influenza in the community. Arch Intern Med, 2001. 161: p. 2116-22*
6. *Boivin G, Hardy I, Tellier G, Maziade J. Predicting influenza infections during epidemics with use of a clinical case definition. Clin Infect Dis. 2000;31(5):1166-1169*
7. *Monto AS, Gravenstein S, Elliott M, Colopy M, Schweinle J. Clinical signs and symptoms predicting influenza infection. Arch Intern Med. 2000;160:3243-3247*
8. *Carrat F, et al., Evaluation of clinical case definitions of influenza: detailed investigation of patients during the 1995-1996 epidemic in France. Clin Infect Dis, 1999. 28(2): p. 283-90*
9. *Govaert Th, Dinant GJ, Aretz K, et al. The predictive value of influenza symptomatology in elderly people. Fam Pract. 1998;15:16-22*
